# *Schistosoma japonicum* Soluble Egg Antigens Facilitate Hepatic Stellate Cell Apoptosis by Downregulating Akt Expression and Upregulating p53 and DR5 Expression

**DOI:** 10.1371/journal.pntd.0003106

**Published:** 2014-08-21

**Authors:** Jianxin Wang, Feifan Xu, Dandan Zhu, Yinong Duan, Jinling Chen, Xiaolei Sun, Xue He, Pan Li, Wei Sun, Jinrong Feng

**Affiliations:** 1 Laboratory Medicine Center, Affiliated Hospital of Nantong University, Nantong, Jiangsu, People's Republic of China; 2 Department of Pathogen Biology, School of Medicine, Nantong University, Nantong, Jiangsu, People's Republic of China; Monash University, Australia

## Abstract

**Background:**

The induction of hepatic stellate cell (HSC) apoptosis has potential as a potent strategy to diminish the progression of liver fibrosis. Previous studies have demonstrated the ability of soluble egg antigens (SEA) from schistosomes to inhibit HSC activation and to induce apoptosis in vitro. In this study, we aimed to explore the mechanism of SEA-induced apoptosis in HSCs.

**Methodology/Principal Findings:**

In this study, we found that SEA could upregulate p53 and DR5 and downregulate the p-Akt. The apoptosis of HSCs induced by SEA could be reduced in HSCs that were treated with p53-specific siRNA and in HSCs that were treated with DR5-specific shRNA. In addition, GW501516, which enhances the expression of Akt, could also decrease the SEA-induced HSC apoptosis. We also found that the increased expression of p53 and DR5 induced by SEA through Mdm2 were reduced by GW501516.

**Conclusions/Significance:**

Our data suggest that SEA can induce HSC apoptosis by downregulating Akt expression and upregulating p53-dependent DR5 expression.

## Author Summary

Schistosomiasis, caused by schistosomes, is one of the most prevalent parasitic infections, and often results in chronic hepatic fibrosis. The induction of HSC apoptosis reverses schistosome-induced hepatic fibrosis. Previous studies have demonstrated that soluble egg antigens (SEA) from schistosomes could inhibit HSC activation and induce apoptosis in vitro. Here, we found that SEA could induce apoptosis in LX-2 cells by upregulating p53 and DR5 and downregulating p-Akt. SEA-induced HSC apoptosis could be decreased by p53-specific siRNA or DR5-specific shRNA. In addition, GW501516, which enhances the expression of Akt, could also reduce the HSC apoptosis induced by SEA. Our data suggest that SEA can induce HSC apoptosis by downregulating Akt expression and upregulating p53-dependent DR5 expression.

## Introduction

Hepatic fibrosis, which is a common result of chronic liver injury, is characterized by the abnormal deposition of extracellular matrix (ECM) proteins [1]. The central pathogenic event of liver fibrosis is the activation of hepatic stellate cells (HSCs) [2]. In the progression of liver fibrosis, quiescent HSCs are often activated by a variety of etiological factors and are transdifferentiated into a myofibroblast-like phenotype to produce high amounts of ECM and to secrete proinflammatory and profibrogenic cytokines [3]. Thus, the removal of activated HSCs is the primary hepatic fibrosis treatment strategy. Many recent studies have focused on the apoptosis of activated HSCs, indicating that this apoptosis mainly contributes to the reversal of hepatic fibrosis [4], [5]. Therefore, the induction of apoptosis in activated HSCs may be an effective hepatic fibrosis treatment strategy.

Schistosomes are among the most common causes of hepatic fibrosis in schistosomiasis-endemic areas. Inflammatory granuloma is the initiation factor in the development of schistosome-induced fibrosis [6], [7]. Some researchers have recently focused on the functions of certain schistosome molecules from schistosome eggs [8], [9]. Eggs and soluble egg antigens (SEA, major complex mixtures that are isolated from schistosome eggs) have anti-fibrotic effects on activated HSCs and play direct anti-inflammatory roles in the innate immune response [10]–[12]. More importantly, Anthony et al. [10], [11] have reported that both *Schistosoma mansoni (S. mansoni)* and *Schistosoma japonicum (S. japonicum)* eggs suppress the activation of HSCs and lead to the downregulation of fibrosis-associated genes. In addition, SEA from *S. mansoni* induces the apoptosis of T helper lymphocytes during murine schistosome infection [13]. Our previous studies have also suggested that SEA from *S. japonicum* inhibits the activation of the human HSC cell line LX-2 and the primary HSCs from *S. japonicum*-infected mice through peroxisome proliferator-activated receptor γ (PPARγ) and the transforming growth factor β (TGFβ) signaling pathway [14]. SEA can also induce apoptosis in LX-2 cells through the caspase-dependent pathway [14]. In this study, we further attempted to explore the role of Akt and p53 in SEA-induced apoptosis in LX-2 cells and aimed to determine the mechanism of SEA-induced HSC apoptosis.

## Materials and Methods

### Cell culture and materials

The immortalized human hepatic stellate cell line LX-2 was purchased from Xiang Ya Central Experiment Laboratory (China) and cultured in Dulbecco's Modified Eagle Medium (DMEM, Gibco, USA) supplemented with 10% fetal bovine serum (FBS, Invitrogen, USA) in a humidified incubator at 37°C with 5% CO_2_. SEA of *S. japonicum* was obtained from Jiangsu Institute of Parasitic Diseases (China). Primary antibodies for mouse double minute protein 2 (Mdm2), total-Akt (T-Akt) and p53 were purchased from Santa Cruz Biotechnology (USA). Primary antibodies for caspase-3 and phospho-Akt (Ser^473^, p-Akt) were purchased from Cell Signaling Technology (USA). The primary antibody for glyceraldehyde phosphate dehydrogenase (GAPDH) was provided by Goodhere (China). All of the secondary antibodies were obtained from Santa Cruz Biotechnology (USA). In addition, GW501516 (Santa Cruz, USA), which is a potent PPARβ/δ agonist, was also used to activate Akt signaling [15].

### RNA interference

Small-interfering RNA (siRNA) against p53 and scrambled siRNA were synthesized by GenePharma (China), and their sequences were as follows: the p53-specific siRNA (si-p53) 5′-GACTCCAGTGGTAATCTACTT-3′ and scrambled control siRNA (si-con): 5′-UUCUCCGAACGUGUCACGUTT-3′ [16]. To silence the expression of the p53 gene, LX-2 cells were transfected with si-p53 using Lipofectamine 2000 (Invitrogen, USA) according to the manufacturer's instructions. After transfected for 30 h, the cells were treated with or without the SEA for another 18 h and then harvested for western blot and quantitative real-time polymerase chain reaction (qRT-PCR) assays.

To silence DR5 expression, we transfected shRNA-control (sh-con, Genechem, China) or shRNA-DR5 (sh-DR5, Genechem, China) in LX-2 cells using FuGENE 6 (Promega, USA) according to the manufacturer's instructions. After transfection for 30 h, the cells were treated with or without the SEA for another 18 h and then harvested for western blot and qRT-PCR assays.

### Western blot

The proteins were extracted from the HSCs and were quantified by the Bradford method (Sangon, China). The protein solution was then separated by 10% or 12% sodium dodecyl sulfate-polyacrylamide gel electrophoresis (SDS-PAGE), electrotransferred onto polyvinylidene fluoride (PVDF) membranes (Merck, Germany), and blocked with 5% nonfat dry milk as previously described [17]. The target proteins were incubated overnight with specific primary antibodies and subsequently with horseradish peroxidase (HRP)-conjugated secondary antibodies. The membranes were then visualized using a chemiluminescence kit (Merck, Germany). The intensity of the bands was measured by Image J and normalized to the intensity of GAPDH. The data were then versus those of the control group and analyzed by SPSS software.

### qRT-PCR

The total RNA was extracted from LX-2 cells using the Trizol RNA isolation reagent (Invitrogen, USA) following the manufacturer's protocol. The RNA was then reverse-transcribed into complementary DNA (cDNA) using the RevertAid First-Strand cDNA Synthesis Kit with Oligo (dT) 18 primers (Thermo Fisher Scientific, USA) as previously described [17]. The cDNA products were then used as the templates for qRT-PCR using a SYBR Premix Ex Taq Kit (Takara, Japan) on the Eco Real-Time PCR Sequence Detection System (Illumina, USA). The target gene values were normalized to GAPDH values and expressed as relative fold increases 2^(−ΔΔCt)^ over the non-treated samples.

### Measurement of caspase-3 activity

The activity of caspase-3 was measured using the caspase-3 Activity Assay Kit (Beyotime, China). Following the manufacturer's instructions, a standard curve was generated by measuring the absorbance (A405) of various amounts of standard *p*NA. Then, the LX-2 cells were incubated with the SEA (10 µg/ml) for 18 h, and the proteins were harvested. Acetyl-Asp-Glu-Val-Asp *p*-nitroanilide (Ac-DEVD-*p*NA) was then added to each sample at 37°C for 2 h. The absorbance was determined on an enzyme-linked immunosorbent assay (ELISA) reader (BioTek, USA) and converted to the amounts of *p*NA that were produced in the cells.

### Immunofluorescence analysis

The cells were fixed with 4% paraformaldehyde in PBS for 30 min and permeabilized with 0.1% Triton X-100 for 2 min. After blocking in 10% donkey serum for 1 h at room temperature, the cells were stained for the primary antibodies against p53 (Santa Cruz Biotechnology, USA) or p-Akt (Cell Signaling Technology, USA) and then incubated with the second antibodies. The cells were also stained with Hoechst 33342 (Sigma, USA) for 15 min and examined by confocal laser-scanning or fluorescent microscopy.

### Annexin V-FITC staining

The apoptosis rate was determined by Annexin V-FITC staining (Merck, Germany). After treatment with the SEA (10 µg/ml) for 18 h, the cells were collected and stained with PI followed by Annexin-V for 15 min in the dark at room temperature according to the manufacturer's instructions. The apoptosis was then analyzed by flow cytometry.

### Statistical analysis

All of the data were presented as the mean ± SEM of three independent experiments and were analyzed using the Independent Samples T-test or One-Way ANOVA in SPSS 15.0 to determine the significant differences. *p*<0.05 was considered statistically significant.

## Results

### SEA induces apoptosis in LX-2 cells

**Figure 1:**
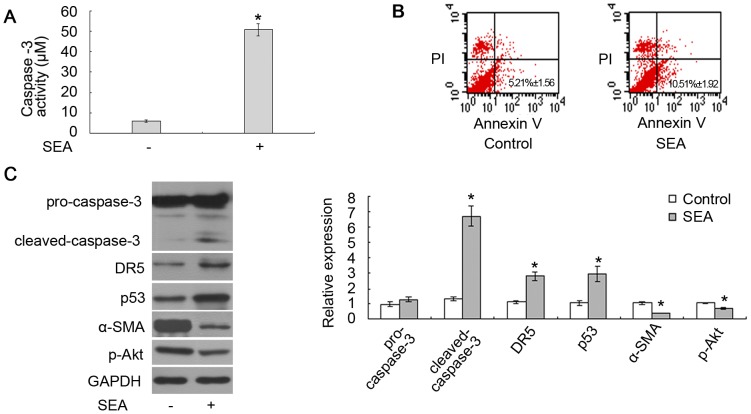
The SEA induced apoptosis in LX-2 cells. (A) The caspase-3 activity of the LX-2 cells that were treated with SEA significantly increased compared to that in the cells without SEA treatment (*p<0.05). The Y-axis represents the activity of caspase-3 in detecting the production of *p*NA. (B) The apoptosis rate of LX-2 cells was analyzed by Annexin-V/PI double staining. SEA could increase the percentage of apoptotic HSCs. (C) The cleaved-caspase-3, DR5 and p53 increased, while the expression of α-SMA and p-Akt decreased in the cells that were treated with SEA. *p<0.05 compared to the control group.

To investigate the effects of SEA on LX-2 cells, these cells were treated with 10 µg/ml SEA for 18 h. As shown in Fig. 1A, the caspase-3 activity of the LX-2 cells significantly increased after being exposed to the SEA for 18 h. The results of Fig. 1B also demonstrated that the percentage of apoptotic cells increased from 5.21%±1.56 (no stimulus treatment) to 10.51%±1.92 (SEA treated) after being exposed to SEA (*p<0.05). We also determined by western blot that the SEA could upregulate the level of cleaved-caspase-3 and downregulate the expression of α-SMA in LX-2 cells (Fig. 1C). In addition, the expression of DR5 and p53 increased and that of p-Akt decreased in LX-2 cells that were treated with the SEA (Fig. 1C).

### SEA induces apoptosis in LX-2 cells by upregulating DR5 expression

**Figure 2:**
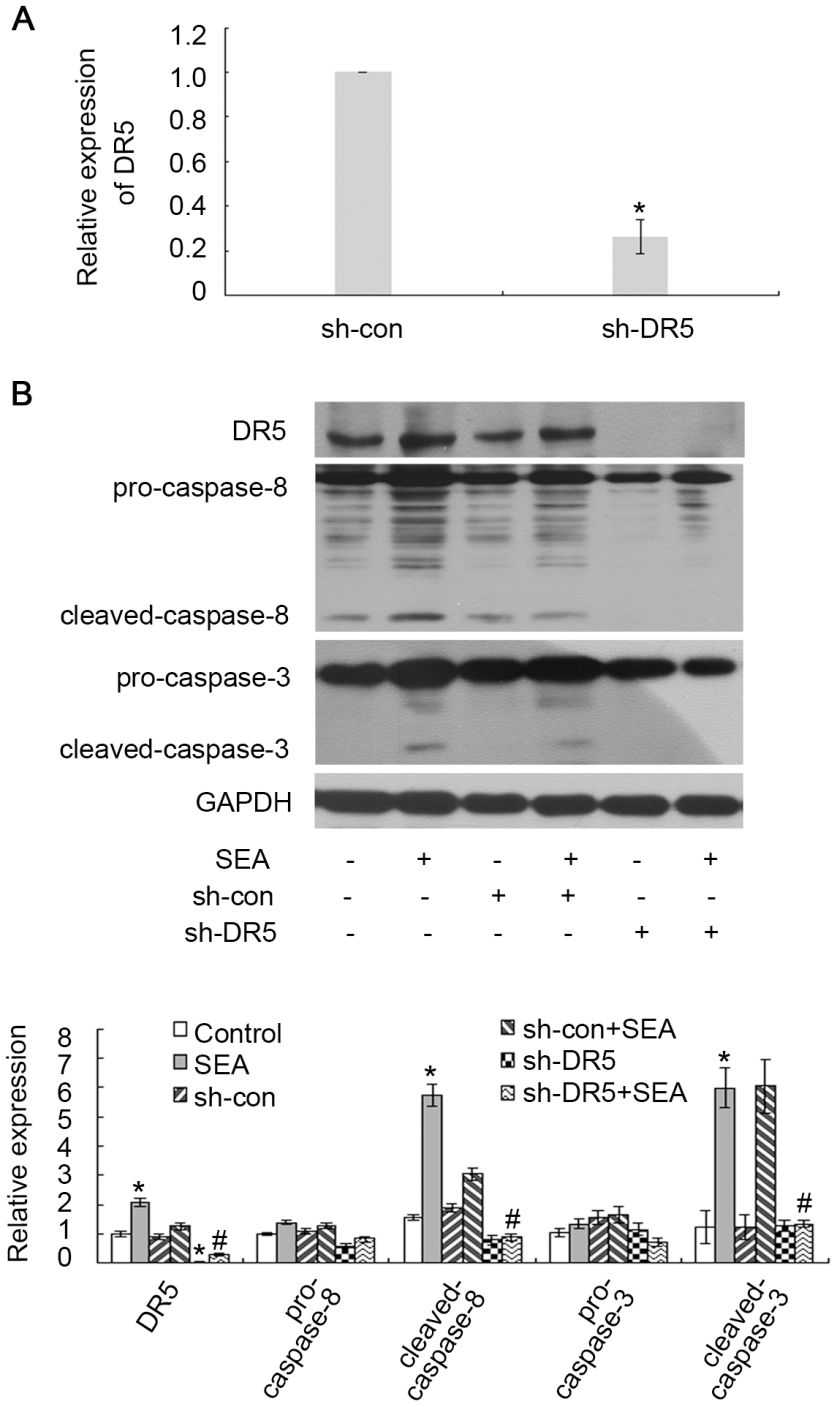
The sensitization of LX-2 cells to SEA-induced apoptosis was associated with DR5 expression. (A) The LX-2 cells were transfected with DR5-target shRNA or scrambled shRNA, and the expression of DR5 in these cells was detected by qRT-PCR. *p*<0.05 compared to the sh-con group. (B) The LX-2 cells were transfected with DR5-target shRNA or scrambled shRNA with or without SEA (10 µg/ml). SEA induces apoptosis in LX-2 cells through the DR5/caspase-8 pathway. *p<0.05 compared to the control group. #*p*<0.05 compared to the group that was treated with SEA.

In our previous studies, we found that SEA could induce apoptosis in LX-2 cells with upregulated cleaved-caspase-8 level [14]. The expression of tumor necrosis factor-related apoptosis-inducing ligand (TRAIL), which is secreted from the SEA-treated HSCs, was upregulated by SEA treatment as determined by ELISA [14]. However, although one of the death receptors (DR5) of TRAIL was upregulated by SEA, no significant differences in the expression of another death receptor (DR4) were found among the SEA-treated HSCs group and the control group [14]. Because TRAIL mediates apoptosis by binding to its death receptors, resulting in the recruitment of the adaptor molecule FADD [18], which activates the signaling pathway of caspase-8 to induce apoptosis, we further evaluated the role of DR5 in the SEA-induced upregulation of cleaved-caspase-8 level. As shown in Fig. 2A, the DR5 shRNA plasmid effectively knocked down the expression of DR5 in LX-2 cells. The SEA upregulated the levels of cleaved-caspase-3 and cleaved-caspase-8 (Fig. 2B). However, this upregulation was reduced in the LX-2 cells that were transfected with the DR5 shRNA plasmid (Fig. 2B). These results further confirm that DR5/caspase-8 signaling sensitizes LX-2 cells to SEA induced-apoptosis.

### SEA induces apoptosis in LX-2 cells by upregulating p53 expression

**Figure 3:**
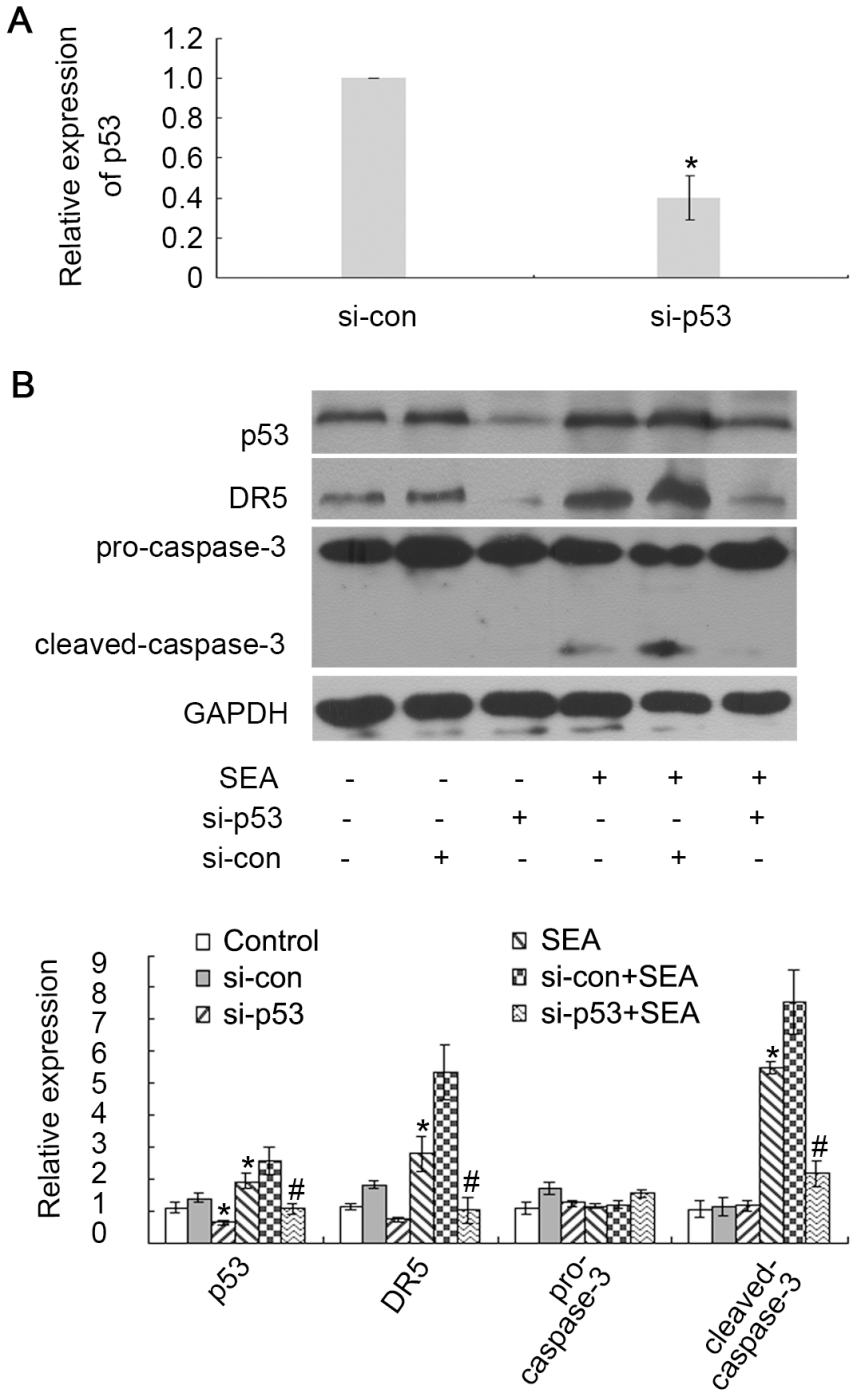
The SEA induced apoptosis in LX-2 cells in a p53-dependent manner. (A) The LX-2 cells were transfected with si-p53 or scrambled siRNA, and the expression of p53 in these cells was detected by qRT-PCR. *p*<0.05 compared to the si-con group. (B) The LX-2 cells were transfected with si-p53 or scrambled siRNA with or without SEA (10 µg/ml) treatment. The DR5 and cleaved-caspase-3 decreased in the SEA-treated LX-2 cells that were transfected with si-p53 compared to that in the SEA-treated LX-2 cells that were transfected with scrambled siRNA. SEA induces apoptosis in LX-2 cells by upregulating p53 expression. *p<0.05 compared to the control group. #*p*<0.05 compared to the group that was treated with SEA.

**Figure 4:**
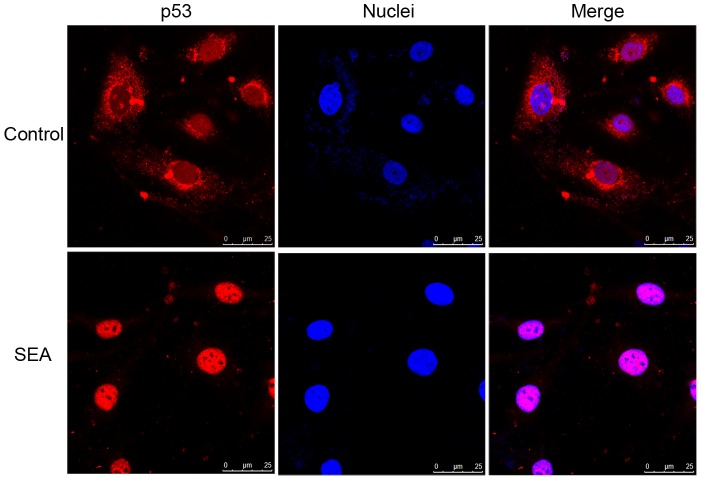
The SEA induced the nuclear accumulation of p53 as detected by immunofluorescence staining. This accumulation was detected in the LX-2 cells that were treated with SEA. The nuclei were stained by Hoechst 33342. The cells were examined by confocal laser scanning microscope.

p53 is an important tumor suppressor that is involved in the cell cycle and cellular apoptosis. To determine whether p53 is involved in SEA-induced apoptosis in LX-2 cells, si-p53 was transiently transfected into LX-2 cells to suppress p53 expression. As shown in Fig. 3A, the expression of p53 was downregulated in the LX-2 cells that were transfected with si-p53 compared to that in the cells that were transfected with si-con. The expression of DR5 was downregulated in the SEA-treated LX-2 cells that were transfected with si-p53 compared to that in the SEA-treated LX-2 cells that were transfected with si-con (Fig. 3B). A similar result for cleaved-caspase-3 is shown in Fig. 3B, indicating that the increase of cleaved-caspase-3 expression level in the cells that were treated with SEA was reversed by si-p53 and not by si-con. In addition, the nuclear accumulation of p53 was detected upon SEA stimulation (Fig. 4). These results indicate that p53 may be involved in the SEA-induced increase in DR5 expression and in SEA-induced apoptosis.

### SEA-induced upregulation of p53 and DR5 expression in LX-2 cells is associated with Akt

**Figure 5:**
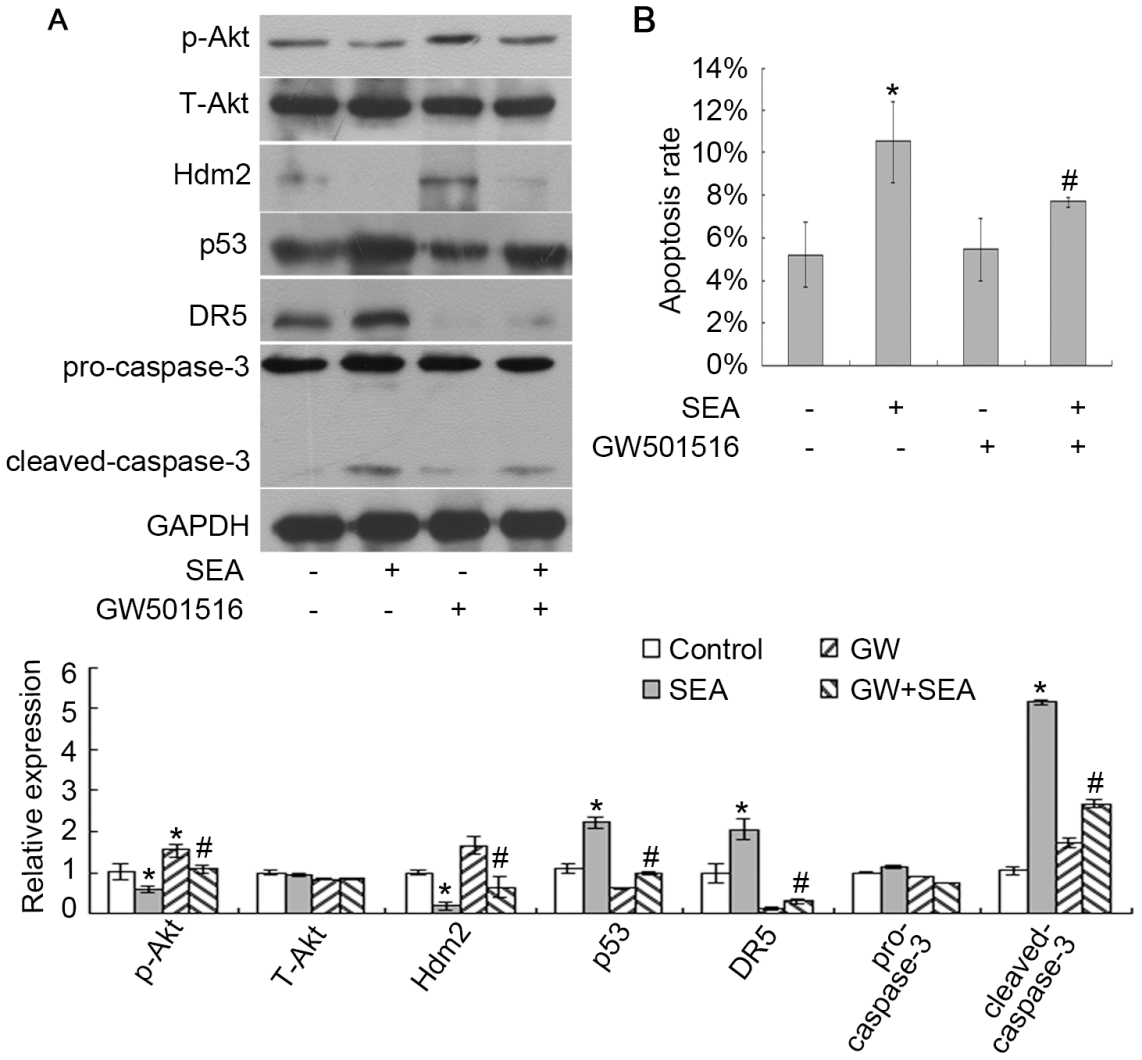
The SEA induced apoptosis in LX-2 cells by downregulating the expression of Akt. (A) The LX-2 cells were treated with GW501516 and SEA (10 µg/ml) and harvested for western blot analysis. The SEA decreased the p-Akt and subsequently increased that of p53 and DR5 in the LX-2 cells through the regulation of Hdm2 expression. *p<0.05 compared to the control group. #*p*<0.05 compared to the group that was treated with SEA. (B) The cellular apoptosis rates were analyzed by Annexin-V/PI double staining. GW501516 partially reversed the SEA-induced apoptosis of the LX-2 cells. *p<0.05 compared to the control group. #*p*<0.05 compared to the group that was treated with SEA.

**Figure 6:**
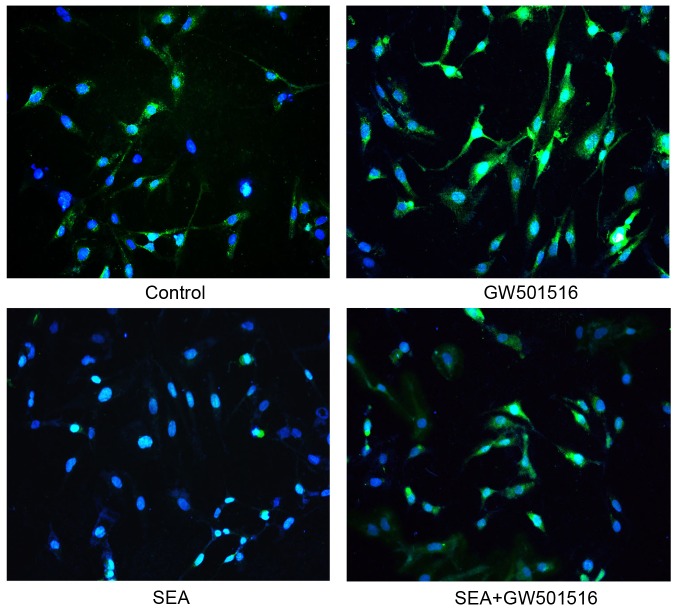
The SEA induced the down-regulation of p-Akt. The images were photographed under fluorescent microscopy (200×). The nuclei were stained by Hoechst 33342. The expression level of p-Akt was downregulated in the cells that were treated with SEA compared to that in the cells with no stimulus.

Akt signaling enhances the expression of Mdm2 and downregulates p53 expression [16]. Because p53 expression was upregulated in the SEA-treated LX-2 cells, we focused on the relationship between Akt and p53 in these cells. First, the level of Akt phosphorylation was downregulated in the SEA-treated LX-2 cells and was reversed when these cells were co-treated with SEA and GW501516 (Fig. 5A). Similar results were found using immunofluorescence analysis (Fig. 6). GW501516 is a potent PPARβ/δ agonist that activates Akt signaling [15]. GW501516 partially reversed the SEA-induced upregulation of p53 and DR5 expression in the LX-2 cells (Fig. 5A). GW501516 could also partially reversed the SEA-induced downregulation of Hdm2 (human homologue of Mdm2) expression in the LX-2 cells (Fig. 5A). In addition, the results of Fig. 5A and 5B showed that the SEA-induced apoptosis in LX-2 cells significantly decreased when the cells were pre-treated with GW501516 and SEA. The results obtained by the method of Annexin-V/PI double staining by flow cytometry (Fig. 5B) indicated that the apoptosis rate of LX-2 cells co-treated with GW501516 and SEA was reduced to 7.69%±0.25, compared to the cells treated with SEA only (10.51%±1.92, #*p*<0.05). Therefore, we suggest that Akt signaling may be reduced in the SEA-treated LX-2 cells, resulting in the activation of p53 and of the downstream apoptosis-associated regulators.

## Discussion

Schistosomiasis caused by schistosomes is one of the most prevalent parasitic infections and often results in inflammatory granuloma and progressive chronic hepatic fibrosis. The induction of HSC apoptosis reverses schistosome-induced hepatic fibrosis. In previous studies, we and other researchers have found that apoptosis phenomenon could be increased in livers with hepatic fibrosis caused by schistosomiasis [14], [19]. We have concluded that some possible components from schistosome could reverse or attenuate liver fibrosis by inducing cell apoptosis [14]. Some studies have recently suggested that the SEA from schistosomes could induce the apoptosis of granuloma T and splenic cells during schistosomal infection [13]. Meanwhile, a recombined protein (rSj16) that was derived from *S. japonicum* induces apoptosis in murine myeloid leukemia cells [8], [13]. In addition, this and a previous study [14] demonstrate that the SEA from *S. japonicum* directly induces the apoptosis of activated HSCs. SEA may play important and interesting roles in the reversible process of hepatic fibrosis. However, the apoptosis induced by SEA seemed to be weak in our studies. Since SEA is a major complex mixture isolated from schistosome eggs, we consider that the apoptosis inducers and the apoptosis inhibitors may be co-existed in SEA. For example, a cytokine-induced apoptosis inhibitor (CIAP) from all the developmental stages of *S. japonicum* could inhibit the caspase activity in human 293T cells [20]. The inhibitor of apoptosis from *S. japonicum* (SjIAP) could also inhibit the caspase activity of 293T cells [21]. Therefore, as a complex mixture, HSC apoptosis was induced by a combination of multiple components of SEA.

The PI3K/Akt pathway is one of the most valuable intracellular signal transduction pathways for suppressing apoptosis, and Akt is the pivotal effector of the PI3K/Akt pathway [22]. For instance, sunitinib (an inhibitor of vascular endothelial growth factor receptor) induces the apoptosis of medulloblastoma tumor cells by inhibiting the STAT3 and Akt signaling pathways [23]. Some studies have recently demonstrated that Akt signaling is also involved in hepatic fibrosis. The inhibition of the Akt/P70S6K pathway could contribute to the reduced expression of collagen genes and the inhibition of HSC proliferation [24]. Furthermore, the suppression of PI3K/Akt signaling can also promote TRAIL-induced apoptosis in LX-2 cells accompanied by the activation of FoxO proteins [25]. In our study, we found that the expression level of p-Akt in LX-2 cells was downregulated by SEA. When Akt signaling was activated by GW501516 in the LX-2 cells, SEA-induced apoptosis could be also reduced by GW501516. These results indicate that the reduction in Akt signaling could contribute to the SEA-induced apoptosis in LX-2 cells.

p53 is the most critical tumor suppressor gene in human cancers and plays a critical role in regulating cell apoptosis [26]. Huang et al. [27] have reported that oridonin induces the apoptosis of HepG2 through the p53, MAPK and mitochondrial signaling pathways. Our data indicated that the expression of p53 increased in the LX-2 cells with SEA treatment and that the silencing of p53 could partially reverse the SEA-induced apoptosis in LX-2 cells.

Some studies have recently demonstrated that activation of Akt signaling could contribute to the regulation of Mdm2, resulting in a reduction in p53 expression [28]–[30]. TRAIL-induced apoptosis in human hepatocellular carcinoma cells is also associated with Notch1 signaling by inhibiting Akt/Hdm2-mediated p53 degradation [16]. Notch1 signaling enhances DR5 expression in a p53-dependent manner to enhance TRAIL-induced apoptosis [16]. TRAIL-induced apoptosis is inhibited in p53-silenced HepG2 cells [16]. These results demonstrate the intimate connection among Akt, Mdm2, p53 and DR5 in apoptosis. In this study, we confirmed that the activation of Akt signaling by GW501516 could reverse the SEA-induced increase in p53 and DR5 expression through the regulation of Hdm2. These results also indicate that silencing p53 expression decreases the expression of DR5 and suggest that SEA induces the apoptosis of LX-2 cells by suppressing Akt signaling and the upregulating p53 and DR5 expression.

In conclusion, we provide a potent mechanism by which SEA induces apoptosis in LX-2 cells. We demonstrate that the inhibition of Akt signaling sensitizes the SEA-induced apoptosis in LX-2 cells by upregulating p53-dependent DR5 expression. Future studies will focus on the main component(s) of SEA in HSC apoptosis.
